# Spatiotemporal Retention of Structural Color and Induced Stiffening in Crosslinked Hydroxypropyl Cellulose Beads

**DOI:** 10.1002/marc.202400755

**Published:** 2024-12-08

**Authors:** Piangtawan Phoungtawee, Taweesak Sudyoadsuk, Torbjörn Pettersson, Daniel Crespy, Anna J. Svagan, Ravi Shanker

**Affiliations:** ^1^ Department of Materials Science and Engineering School of Molecular Science and Engineering Vidyasirimedhi Institute of Science and Technology (VISTEC) Rayong 21210 Thailand; ^2^ Frontier Research Center (FRC) Vidyasirimedhi Institute of Science and Technology (VISTEC) Rayong 21210 Thailand; ^3^ Department of Fibre and Polymer Technology School of Engineering Sciences in Chemistry Biotechnology and Health KTH Royal Institute of Technology Stockholm SE‐100 44 Sweden

**Keywords:** chiral nematic, crosslinker, hydroxypropyl cellulose, self‐assembly, structural colors, Young's modulus

## Abstract

Hydroxypropyl cellulose (HPC) is known for its ability to form cholesteric liquid crystalline phases displaying vivid structural colors. However, these vibrant colors tend to fade over time when the material dries. This issue is a major bottleneck to finding practical applications for these materials. Here this problem is overcome by producing free‐standing, millimeter‐sized HPC structurally colored beads with spatiotemporal color retention, facilitated by a glutaraldehyde crosslinker. By leveraging the well‐known chemically induced stabilization of cholesteric liquid crystalline phases, stable structural colors are achieved for at least three weeks. The presence of glutaraldehyde significantly increases the mechanical stiffness, with Young's modulus rising from 0.3± 0.1 GPa to 1.8± 0.2 GPa. This integrated approach of creating free‐standing photonic HPC beads offers a strategy for developing robust and durable photonic HPC materials with enhanced stability, advancing photonic material applications with spatiotemporal color stability.

## Graphical abstract

This work introduces a strategy to produce glutaraldehyde‐crosslinked hydroxypropyl cellulose (HPC) beads with stable, vivid structural colors retained over weeks. By optimizing crosslinking conditions, these beads achieve color retention, enhanced mechanical properties, and angle‐independent coloration. The study highlights their potential for applications in durable photonic materials, sensors, and aesthetic applications, offering a practical solution to color fading and fragility challenges.

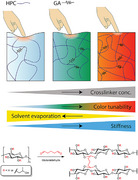

## 1 Introduction

Hydroxypropyl cellulose (HPC) is a derivative of natural cellulose, known for its solubility in water and versatility in applications such as pharmaceuticals,^[^ ^1^, ^2^, ^3^ ^]^ edible films,^[^ ^4^, ^5^ ^]^ and hydrogels.^[^ ^6^, ^7^ ^]^ One of the most intriguing properties of HPC is its ability to form cholesteric liquid crystalline phases, exhibiting vibrant structural colors (SC) at high concentrations (above 55 wt.%).^[^ ^8^, ^9^, ^10^ ^]^ The structural color displayed by HPC can respond to various external stimuli by altering their volume or refractive index. The SC arises from the helical arrangement of HPC molecules, which reflects specific wavelengths of light. The reflected color depends on the pitch (the distance over which a helical structure completes one full turn, determining the wavelength of light reflected and, consequently, the observed color in chiral nematic phases) of the helix, which can be tuned by varying the concentration of HPC. For example, red colors are observed at ≈63 wt.% solid concentration.^[^ ^11^ ^]^ As the concentration of HPC further increases (66‐70 wt.%), the molecules pack more closely, forcing the cholesteric pitch to decrease. This shorter pitch causes a blueshift in the reflected color, and at very high concentrations, it can shift into the ultraviolet range, making the material appear colorless.^[^ ^11^ ^]^ Previous studies have also reported that the structural colors of HPC depend on temperature, which can alter the pitch and affect the perceived structural colors.^[^ ^12^, ^13^ ^]^ Therefore, a significant challenge in utilizing HPC‐based photonic materials is maintaining these structural colors upon drying/solvent evaporation. This phenomenon is similar to issues observed in nature and industrial coatings where controlling evaporation is essential for preserving functional properties.^[^ ^14^, ^15^, ^16^, ^17^ ^]^ For example, plant leaves have waxy cuticles to regulate water loss, and industrial coatings often develop protective skins during drying to maintain their integrity.^[^ ^17^, ^18^, ^19^, ^20^, ^21^ ^]^ Similarly, in HPC, the challenge is to stabilize the cholesteric structure to retain its optical properties. As the cholesteric structures are obtained in hydrated HPC, the structures are fragile and the color disappears when the bead dries at ambient conditions.^[^ ^22^ ^]^ Hence, to achieve a dry bead with a SC, various strategies have been explored, including chemical crosslinking to enhance both optical and mechanical stability in the final dried form. One way to address this issue is to use crosslinking. Since it is known that crosslinking stabilizes the structure by forming covalent bonds between polymer chains and preventing collapse during drying. Physical crosslinking has been attempted by mixing HPC with other polymers such as gelatin^[^ ^23^ ^]^ or poly(ethylene glycol),^[^ ^24^ ^]^ which form hydrogen bonds between the polymer chains. On the other hand, UV crosslinking and chemical crosslinking present a more promising approach.^[^ ^25^, ^26^, ^27^ ^]^ Previous studies have also explored different crosslinkers, such as diisocyanates, diacrylates, orthosilicates, and dialdehydes, including glutaraldehyde (GA).^[^ ^27^, ^28^, ^29^, ^30^, ^31^, ^32^ ^]^ GA, in particular, has shown promise in preserving the cholesteric structure and controlled non‐iridescent colors.

**Figure 1:**
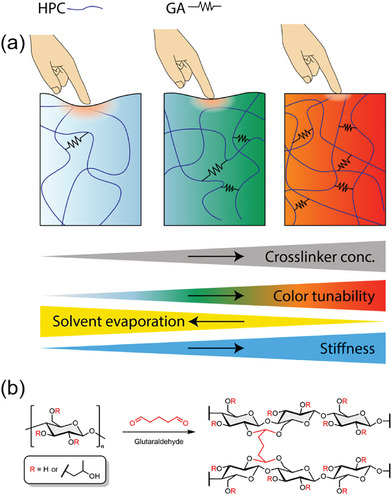
a) Simplified schematics illustrating the effects of GA concentration on HPC. From left to right, the diagram depicts the progression from low to high GA crosslinking concentration. The arrows indicate the corresponding changes in material properties: color tunability, solvent evaporation rate, and stiffness. b) cross‐linking mechanism.

The present work explores a facile one‐step method to produce GA‐crosslinked HPC‐based blue, green, and orange/red structurally colored beads (SCBs). These beads exhibit spatiotemporal retention of their structural colors, meaning they retain color uniformity across the bead (spatial retention) while also maintaining stable colors over several weeks (temporal retention). Additionally, this study includes a non‐crosslinked sample, hereafter referred to as nc‐SCBs, for comparison. The obtained SCBs address both color retention and mechanical robustness simultaneously by varying GA concentrations (**Figure** **1**). Increasing the concentration of GA leads to a higher cross‐link density, thereby restricting the mobility of the polymer chains and resulting in a marked increase in mechanical stiffness. These SCBs demonstrate stable and tunable structural colors that can be retained for at least three weeks, a significant improvement compared to their counterparts nc‐SCBs. Furthermore, at the macro scale, these SCBs show angle‐independent colors, meaning their color does not change when viewed from different perspectives/angles. This property, which tends to improve with higher GA concentrations, is important for applications where consistent color is needed, regardless of the viewing angle or perspective, such as in displays, sensors, and decorative materials. This work proposes a versatile strategy for developing a robust solution to the dual problems of color loss and mechanical weakness in HPC‐based systems by optimizing the cross‐linking conditions.

## 2 Results and Discussion

**Figure 2:**
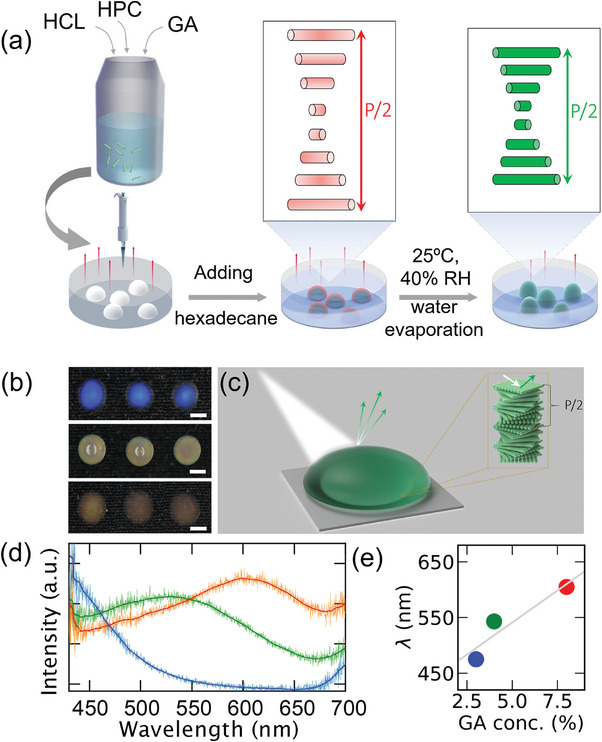
a) Schematic illustration of the preparation of SCBs, including self‐assembly and drying, using hexadecane to slow down evaporation. b) Digital photographs of millimeter‐sized blue, green, and red SCBs, with multiple samples demonstrating process consistency. The scale bars in (b) are 4 mm. c) Schematic illustration shows the interaction of light with the photonic HPC bead. The bead's selective reflection in the visible spectrum generates a green structural color, which is determined by the chiral pitch of the SCBs. d) Reflectance spectra of HPC beads showing reflectance peaks, corresponding to blue, green, and orange colors, respectively. e) Peak maxima wavelength as a function of GA concentration, demonstrating a redshift with increasing concentrations. Data represent the mean values from three measured spectra for each sample, as shown in b) and Figure S3 (Supporting Information).

**Figure** **2**a shows a simplified schematic illustration of the self‐assembly process of HPC‐GA solution into freestanding SCBs. A precise amount of HPC‐GA solution (details in the experimental Section) was placed onto a hydrophobic polystyrene petri dish and left drying with an open lid. To slow down the evaporation rate, a thin layer of hexadecane covered the droplets, and the SCBs were self‐assembled at 25 °C and 40% relative humidity. The presence of hexadecane slows down evaporation, preventing the formation of a coffee‐ring by maintaining a more uniform evaporation flux. As the water evaporated, the material was simultaneously cross‐linked, transitioning from a liquid state to a solid hemispherical SCBs with consistent sizes and shapes. We prepared SCBs using different weight concentrations of GA. HPC can be crosslinked by acetalization with GA in the presence of an aqueous acidic solution at room temperature. Figure 2b shows digital photographs of HPC‐GA photonic beads with blue, green, and red SC, corresponding to 3%, 4%, and 8% GA concentrations, respectively, under normal diffuse ambient light, with reflected SC shifting gradually from blue to orange/red with increasing GA content. The HPC beads were left to dry for 6 days (Figure S1, Supporting Information). This structural coloration arises from the helical arrangement of HPC molecules, where the gradual twist in molecular orientation leads to a photonic bandgap. The resulting color depends on the chiral pitch, as illustrated in Figure 2c. For comparison, control samples, nc‐SCBs, were prepared by the same process but without adding the crosslinker. For the nc‐SCBs, we observed a complete disappearance of structural coloration within 2 days. This was attributed to the significant water loss that, as shown previously, resulted in a changed pitch value that shifted toward the UV region.^[^ ^33^ ^]^ We also carried out experiments with other crosslinkers such as borax and divinyl sulfone, as shown in Table S2 (Supporting Information). The hydroxyl group of HPC can be crosslinked with an aqueous borax solution at relatively low temperatures and deprotonated hydroxyl groups of cellulose in alkaline solutions can react with divinyl sulfone group to yield a crosslinked material.^[^ ^34^, ^35^, ^36^, ^37^ ^]^ Borate species react with hydroxyl groups and vicinal diols of the glucose units in HPC. However, when borax was employed as the crosslinker, colors disappeared after 2 h due to poor crosslinking, which we think is attributed to the limited solubility of borax in water (≈32 g L^−1^ at 25 °C).^[^ ^38^ ^]^ In the case of divinyl sulfone (DVS), colors from samples D1‐2 disappeared after 2 h and after 48 h for sample D3 due to the lower activity of DVS compared with GA (Figure S2, Supporting Information). Samples D4‐D5 did not display colors, likely due to the large amount of DVS hindering cholesteric arrangement. While borax and DVS showed limited effectiveness in maintaining structural colors, GA proved to be the most efficient crosslinker for preserving the cholesteric structure and the associated photonic properties of SCBs.^[^ ^29^ ^]^ During solvent evaporation, the system undergoes complex structural changes governed by dynamics induced by the inclusion of crosslinking agents. GA crosslinks with HPC, forming acetal bonds that hinder the movement of polymer chains and increase solution viscosity. This restricts the movement of HPC molecules, leading to kinetic arrest in agreement with previous studies.^[^ ^39^, ^40^ ^]^ As crosslinking density and HPC concentration increase, stable, free‐standing SCBs are formed. Reflectance spectra were measured to quantitatively analyze the structural colors of SCBs. The spectra show broad peaks at $\lambda_{max}$ = 475, 543, and 605 nm, which correspond to blue, green, and orange SC, respectively (Figure 2d). Plotting the peak maxima wavelength against GA concentration reveals a clear trend: the wavelength increases with higher GA concentrations (Figure 2e). This shows that as GA concentration increases, the chiral pitch, *p*, of the HPC structure increases, causing a redshift in the reflected wavelength. The chiral nematic pitch, increased because the GA crosslinked the HPC molecules, thereby increasing the distance between HPC molecules. Consequently, the change in pitch affects the wavelength‐selective reflection, which can be estimated by the de Vries equation:^[^ ^41^ ^]^

(1) $$\;\lambda_{r}=n\cdot p\cdot \cos\left(\theta \right)$$

where n is the mean refractive index, θ is the angle of incidence (with 0° being normal incidence), and p is the pitch of the helical structure as defined above.

We also observed that increasing GA concentration leads to a broader spectral width of the reflected peak (Figure 2d), indicating an increase in disorder within the HPC mesophase. This phenomenon is similar to observations in opal photonic crystals, where increased disorder results in significant changes in optical properties, such as broadening of the photonic band gap and enhancement of diffuse scattering, due to changes in the photon density of states.^[^ ^42^, ^43^, ^44^ ^]^ The cause of this broadening in our HPC‐GA system likely results from such disorder‐induced changes, similar to those observed in disordered photonic structures. As the level of disorder increases, more states may be introduced near the edges of the partial band gap, leading to a broader and shallower dip in the density of states. This increase in packing defects and structural disorder with higher GA content supports the widening of the spectral width, as the mesophase becomes less ordered and more heterogeneous.

**Figure 3:**
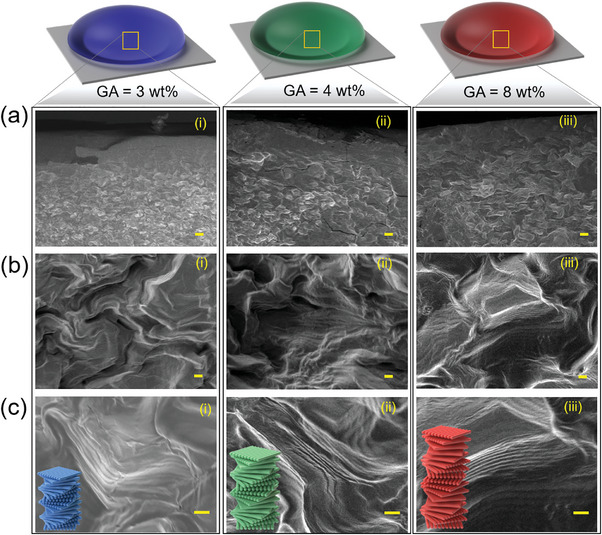
Cross–sectional SEM micrographs of blue, green, and orange SCBs at 25 °C, 40% RH, and imaged at a) shell of the bead b) core of bead (low magnification), and c) core of bead (high magnification). Inset shows cartoons to illustrate the change in cholesteric pitch with increasing the GA concentration. The scale bar represents 10 µm in panel (a) and 1 µm in panels (b) and (c).

Using polarized optical microscopy (POM), we examined the microstructure of SCBs to detect ordered domains. The SCBs were analyzed under cross‐polarizers and rotated at angles of 45°, 90°, 135°, and 180°. The internal structure of the beads appeared grainy, independent of the rotation angle, implying that the cholesteric domains were randomly oriented and exhibited some buckling (Figures S4–S6, Supporting Information).^[^ ^31^ ^]^ Upon close inspection, the dark edges or rim observed under the microscope in our hemispherical SCBs could be due to a combination of factors. For example; the variation in light incidence angles, as detected using microscope objectives with different numerical apertures (NA), plays a role in this effect, with higher NA lenses revealing more of the reflected light variation.^[^ ^45^ ^]^ Additionally, the uniformly black appearance of the bead edges could suggest low birefringence and a lack of cholesteric ordering, both contributing to the observed optical characteristics. To observe the internal microstructure, we performed scanning electron microscopy (SEM) on cross‐sections of blue, green, and orange SCBs, which were freeze‐cut by cooling in liquid nitrogen and then sectioned with a scalpel. The SEM images revealed distinct differences in polydomain structures at the edge (surface) and in the bulk (core) of the HPC beads. The cross‐sections showed a skin layer with a thickness of ≈50 µm (**Figure** **3**a). This skin layer potentially slowed down the evaporation rate of water from the bulk hydrated cholesteric phase.^[^ ^32^, ^46^ ^]^ Additionally, the formation of a skin layer during drying, may suppress iridescence. A similar effect was noted in HPC structures where compression influenced optical properties.^[^ ^32^ ^]^ This finding is consistent with our observation in micro reflection spectroscopy measurement of SCBs showing blue shift at the edges, as shown in Figure S7 (Supporting Information). Higher magnification images highlighted the characteristic Bouligand structure of the cholesteric polydomain (Figure 3b,c).^[^ ^47^, ^48^ ^]^ We measured the pitches of these structures from SEM images, which correspond to the observed colors: beads with blue, green, and orange colors displayed pitches of 301 ± 40 nm, 343 ± 47 nm, and 462 ± 72 nm, respectively. By plotting the cholesteric pitch against the reflectance wavelength, we confirm the clear correlation and predictability of color shifts based on the pitch values observed in the SEM analysis (Figure S8, Supporting Information).

**Figure 4:**
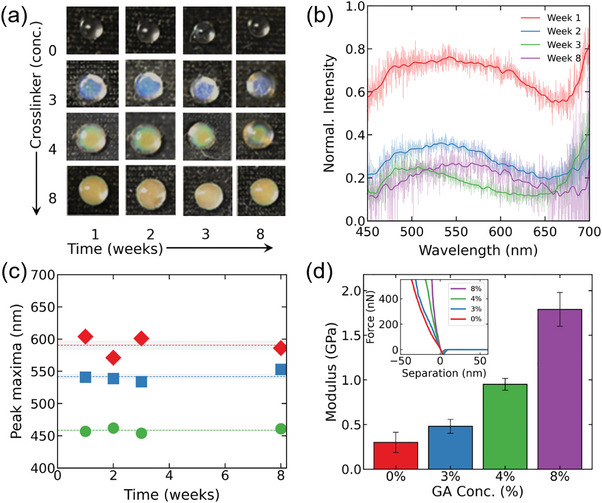
a) Digital photographs of SCBs and nc‐SCBs with varying crosslinker concentrations (0%, 3%, 4%, and 8%) over different time periods (1, 2, 3, and 8 weeks), demonstrating color stability. b) Raw reflectance spectra of SCBs beads with 4% GA concentration over different time periods (1, 2, 3, and 8 weeks), showing stable reflectance peaks. Raw spectral data (light lines) with smoothed data (bold lines) for visual clarity. Smoothing highlights trends without altering the underlying noise. c) Time evolution of reflectance peak maxima against time for different crosslinker concentrations, indicating stable peak positions with minor deviations over 8 weeks. d) Variation in Young's modulus as a function of varying crosslinker concentration in SCBs and nc‐SCBs. The inset shows typical force versus separation curves obtained from nanoindentation measurement.

We next evaluated the long‐term stability of SCBs. Crosslinked SCBs were placed at 28 °C and 75–85% relative humidity. **Figure** **4**a shows digital photographs of SCBs with various concentrations of crosslinker (0%, 3%, 4%, and 8%) over time periods of 1, 2, 3, and 8 weeks. The digital photographs demonstrate that the beads retained their blue, green, and orange colors over the course of 8 weeks, indicating good color stability, except for the nc‐crosslinked SCBs which did not display a SC in the visible color range.

To further analyze this stability, we measured the reflectance spectra of the SCBs and nc‐SCBs. Figure 4b (Figure S9, Supporting Information for nc‐SCBs) shows the raw reflectance spectra for the SCBs beads with 8% crosslinker concentrations up to 2 months (Figure S9, Supporting Information for 0%, 3%, and 4% GA), which demonstrate that the peak reflectance wavelengths remain stable across over time. The purpose of these experiments was to investigate whether minor changes in the cholesteric pitch could occur over time due to the migration of crosslinkers or subtle molecular rearrangements. To take a closer look at the stability, we plotted the peak maxima wavelengths against time for each crosslinker concentration (Figure 4c). The results show that the peak positions (457, 541, and 604 nm initially) remain consistent with minor deviations over the 8‐week period. This consistency in peak wavelength positions confirms the color stability of the crosslinked SCBs over time. The addition of glutaraldehyde crosslinks the HPC chains, chemically locking the cholesteric pitch at specific distances. Kinetic trapping occurs because the crosslinking process immobilizes the cholesteric structure, preventing significant structural rearrangement over time.

We also investigated the mechanical properties of the HPC beads as a function of cross‐linker content. Nanoindentation atomic force microscopy was used to investigate the elastic modulus of the HPC beads with various crosslinker concentrations, as illustrated in Figure 4d. The results showed significant enhancement in the elastic moduli with increasing concentrations of the crosslinker. For dry nc‐SCBs, the elastic modulus was recorded at 0.3 ± 0.1 GPa. This value is expected to decrease further upon hydration, which is typical for hydrogel systems, where the modulus generally decreases with increasing water content due to the plasticizing effect of water. Similar trends have been observed in cellulose nanofiber hydrogels, where the elastic modulus tends to decrease upon wetting, reflecting the reduction in solid content and increased flexibility of the hydrated network.^[^ ^49^, ^50^ ^]^ Introducing a 3% crosslinker resulted in a slight enhancement, with the elastic modulus measured at 0.5 ± 0.08 GPa. A more substantial increase was observed at higher crosslinker concentrations, with SCBs crosslinked with 4% GA exhibiting an elastic modulus of 1 ± 0.07 GPa, and the beads crosslinked with 8% GA reaching 1.8 ± 0.2 GPa. Overall, the findings indicate that adding more GA leads to stiffer beads. This demonstrates the potential for fine‐tuning the mechanical properties of HPC beads through controlled crosslinker addition. Similar to the optical response, the mechanical properties of the SCBs are significantly influenced by the crosslinker. This behavior is consistent with other studies where the mechanical properties of composite films were controlled through the use of specific crosslinking agents or additives.^[^ ^51^, ^52^, ^53^ ^]^

**Figure 5:**
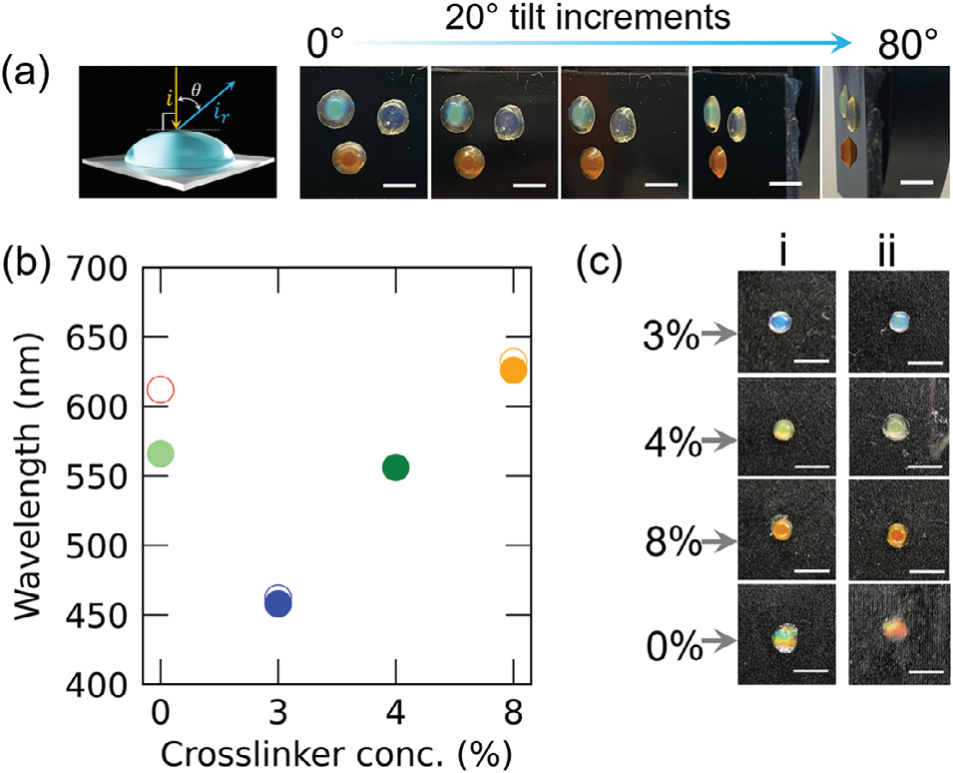
Structural color stability and noniridescent colors. a) Non‐iridescence of SCBs: A schematic of the measurement setup is shown to the left, followed by photographs of SCBs at different rotation angles under ambient diffuse light, showing non‐iridescence. b) Plot of reflectance changes at $\lambda_{max}$ versus crosslinker concentration before (colored solid circles) and upon 5 min of water immersion (colored hollow circles) showing SC retained except without crosslinker SCBs. c) digital photographs showing SC optical comparison of SCBs before immersion (i) and after immersion (ii) showing only SCBs without crosslinker exhibiting a significant change in SC from green to reddish‐orange. The scale bar in (a) is 4 and 5 mm in (c).

To investigate angle‐independent coloration, we studied blue, green, and orange SCBs by taking angle‐dependent photographs in diffuse scattering mode, with the detector fixed and the samples rotated under ambient diffuse light from 0° to 80° (**Figure** **5**a). The SCBs exhibited angle‐independent, matte coloration, with the strongest effect observed in the beads with the highest crosslinker concentration. Previous studies have shown that crosslinker‐induced disorder contributes to angular independence in photonic films.^[^ ^31^ ^]^ In our case, the hemispherical geometry of the beads further enhances this effect which was also observed in studies on assemblies of spherical nanoparticles and polymer microbeads.^[^ ^45^, ^54^, ^55^ ^]^ The curvature ensures that, regardless of the viewing angle, some regions of the chiral nematic structure are always optimally oriented to reflect light toward the detector. The combination of increased crosslinker concentration and non‐planar bead geometry creates a unique system where both factors synergistically contribute to the observed optical properties. These findings suggest that hybrid samples, combining crosslinked and non‐crosslinked regions, could serve as versatile optical indicators, maintaining color stability under normal conditions while providing detectable color changes in response to specific environmental stimuli.

To demonstrate the stability of SC in an aqueous environment, blue, green, and orange crosslinked SCBs were immersed in water for 5 min. As shown in Figure 5, the reflectance wavelength maxima and digital photographs (Figure 5b,c; Figure S10, Supporting Information) demonstrate that crosslinked SCBs retained their SC upon immersion in water.

In contrast, the control beads without the GA crosslinker, prepared at an HPC concentration of 60%, reflected a green color due to their cholesteric pitch. Upon immersion, these beads displayed a significant red shift, losing their initial color integrity. The glutaraldehyde crosslinker prevents structural rearrangement upon exposure to water. In the non‐crosslinked HPC beads, the $\lambda_{max}$ shifted from 568 nm (greenish color) to 612 nm (red color), resulting in a shift of $\Delta \lambda_{max}$ = 44 nm. This observed shift is primarily due to swelling in water with changes in the cholesteric pitch contributing to the redshift. Additionally, using the effective medium approximation for the two‐phase system (air‐HPC to water‐HPC), the effective refractive index increases may also increase slightly upon water ingress, contributing to the redshift. However, compared to refractive index changes, swelling remains the dominating mechanism behind this redshift.

The crosslinked beads maintain their SC because the crosslinking maintains the cholesteric order. This property is valuable in applications where consistent color stability is required, such as in static displays or decorative materials. Conversely, non‐crosslinked or hybrid designs could be used in colorimetric sensors where color changes indicate specific analytes.

## 3 Conclusion

Our study demonstrates that glutaraldehyde crosslinking of HPC beads successfully addresses the dual challenges of color retention and mechanical strength. The crosslinked HPC beads retain their vibrant colors over extended periods, showing remarkable resistance to water exposure. This performance significantly suroutpaces that of non‐crosslinked counterparts. The results indicate that increasing GA concentration leads to a higher cross‐link density, resulting in improved stiffness of the HPC SCBs beads. This enhancement in mechanical properties is directly correlated with the concentration of the crosslinker, demonstrating a clear path for fine‐tuning the properties of HPC beads to meet specific application needs. Notably, the elastic modulus increased from 0.3 to 1.8 GPa as the crosslinker concentration was increased from 0% to 8%, reflecting a significant improvement in mechanical behavior while maintaining the structural coloration. Overall, our work establishes a novel approach for creating HPC beads that combine durability with aesthetic appeal. This method offers a promising avenue for the development of new materials that require both mechanical strength and vibrant coloration, expanding the potential for innovative applications in various fields, including sensors, coatings, and decorative materials.

## 4 Experimental Section

### Materials

Hydroxypropyl cellulose (dry powder, HPC grade SSL‐SFP, *M*_w_ = 40 000 g mol^−1^, Nippon Soda Co., Ltd), glutaraldehyde (GA, 50 wt.% aqueous solution, TCI chemical), hydrochloric acid (HCl, >36.5%, Carlo Erba), and hexadecane (>98%, TCI chemical), sodium hydroxide (NaOH, 98%, Carlo Erba), divinyl sulfone (DVS, 96%, TCI chemical) and di‐sodium tetraborate decahydrate (borax, 99.5%, QREC) were used as received. Deionized water was used throughout this work.

### Crosslinking of HPC Beads

Known amounts of 0.5 m aqueous HCl solutions (440 or 520 or 540 mg) were mixed with an aqueous solution of GA (60, 80 or 160 mg) in a 10 mL glass vial. HPC powder (400 mg) was subsequently added to the acidic solution to produce a 40 wt.% HPC solution. The mixture was stirred at 25 °C for 15 min and then heated at 45 °C for 30 min. The various formulations are shown in Table S1 (Supporting Information).

Around 7.5–7.9 mg of HPC solution was dropped in a polystyrene petri dish (35 mm), to which 1 mL hexadecane was added. The petri dish was covered with a lid and placed in a temperature and humidity chamber at 40% RH at 25 °C.

In the case of HPC crosslinked by borax, HPC powder (325 mg) was dissolved in a mixture of 175, 100 or 87.5 mg of a solution of aqueous borax (30 g L^−1^) and 0 or 75 or 87.5 mg water and then centrifuged 5 times at 10 000 rpm for 30 min at 25 °C. In the case of HPC crosslinked by divinyl sulfone, HPC powder (325 mg) was dissolved in a mixture of 55 mg of a 0.1 m NaOH aqueous solution and water (120,105, 100, 40, and 0 mg). Divinyl sulfone was subsequently added (0 or 15 or 20 or 80 or 120 mg) and the solution was then centrifuged 5 times at 10 000 rpm for 30 min at 25 °C. Around 7.5–7.9 mg of HPC solution was dropped in a polystyrene petri dish (35 mm). The various formulations are listed in Table S2 (Supporting Information).

For the water immersion and color stability test, control beads without the GA crosslinker were prepared at an HPC concentration of 60%. At this concentration, the beads reflected a green color due to their cholesteric pitch. Upon immersion in water, these beads exhibited a significant red shift, losing their initial color.

### Analytical Tools

Photographs of the HPC beads were taken under white light with a digital camera (Nikon D750 equipped with a 105 mm Nikkor Microlens) and a ruler with millimeter‐scale marking in a black photo box. Reflectance spectra of HPC SCBs beads were recorded with a fiber optic spectrometer (USB4000, Ocean Optics), using a 600 µm core optical fiber (UV–vis Optical Fiber, Ocean Optics). A standard white diffuser (Spectralon Diffuse Reflectance Standards, Labsphere) was used as a reference. All measurements were carried out in triplicate. In our micro‐spectroscopic setup, optical microscopy images and reflectance spectra were captured using a custom‐built micro‐spectroscopy system. A 50 W LED/halogen lamp provided illumination for the samples, which were examined under a Motic BA310 Pol microscope, connected to a Moticam ProS5 Lite camera. The reflected light was channeled from the camera port through an optical fiber into a Thor Labs CCS 200 M spectrometer. A 40x magnification objective lens with a numerical aperture (NA) of 0.65 was employed for collecting the spectral data. To ensure accuracy, the reflectance data were referenced against a Thor Labs silver mirror (ME1‐P01).

Polarized optical micrographs (POM) were taken with a stereomicroscope (Zeiss, Discovery V12) equipped with a cross polarizer and a rotating stage.

Cross‐sectional micrographs of the HPC beads were taken with a scanning electron microscope (SEM, JSM‐7610F, JEOL). The HPC beads were placed on aluminum substrates which were placed in liquid nitrogen and cut with a scalpel. Cross‐sections were then mounted on aluminum plates using conductive carbon tape and coated with platinum for 2 min.

Indentation was employed with AFM to determine the mechanical properties. The AFM was performed using a Multimode 8 with an EV scanner (Bruker, Santa Barbara, CA, USA) using an RTESPA‐150 cantilever (Bruker, Camarillo, CA) having a nominal spring constant of 5 N m^−1^ and a nominal tip radius of 8 nm. The type of cantilever was carefully selected to avoid non‐linearity in the detector response.^[^ ^56^ ^]^ The inverse optical lever sensitivity was determined by pressing the cantilever toward a clean silica wafer, and the spring constant was obtained using the built‐in thermal tune function.^[^ ^57^ ^]^ Indentation curves were measured at multiple locations on the sample using a 500 nm ramp size and a scan rate of 1 um s^−1^ with a trigger value of ≈600 nN. This results in indentation into the samples in the range of 5 to 40 nm depending on the material properties of the sample (Figure 4d). Young's modulus was calculated by fitting the linearized Hertz model to force versus separation curves in the force range of 5 to 400 nN, using a poisons ratio of 0.3 and tip radii of 20 nm.^[^ ^58^, ^59^ ^]^ The conversion of the raw data into force versus indentation curves^[^ ^60^ ^]^ and the Hertz fitting were done using the software AFM Force IT v3 (ForceIT, Sweden).

## Conflict of Interest

The authors declare no conflict of interest.

## Supporting information



Supporting Information

## Data Availability

The data that support the findings of this study are available from the corresponding author upon reasonable request.
