# Supplementary material for: Spatiotemporal Retention of Structural Color and Induced Stiffening in Crosslinked Hydroxypropyl Cellulose Beads
Source: Macromol Rapid Commun. 2024 Dec 8;46(5):2400755. doi: 10.1002/marc.202400755 (PMC11884224; doi:10.1002/marc.202400755)
Supplement: Supplementary file 1 — Supporting Information [file MARC-46-2400755-s001.docx]

**Supporting information**

**Spatiotemporal Retention of Structural Color and Induced Stiffening in Crosslinked Hydroxypropyl Cellulose Beads**

Piangtawan Phoungtawee^1^, Taweesak Sudyoadsuk^2^, Torbjörn Pettersson^3^, Daniel Crespy^1^, Anna J. Svagan^3*^ and Ravi Shanker^3*^

^1^Department of Materials Science and Engineering, School of Molecular Science and Engineering, Vidyasirimedhi Institute of Science and Technology (VISTEC), Rayong 21210, Thailand.

^2^Frontier Research Center (FRC), Vidyasirimedhi Institute of Science and Technology (VISTEC), Rayong, Thailand 21210

^3^Department of Fibre and Polymer Technology, School of Engineering Sciences in Chemistry, Biotechnology and Health, KTH Royal Institute of Technology, Stockholm SE-100 44, Sweden

**Keywords:** structural colors, hydroxypropyl cellulose, Young’s modulus, self-assembly, crosslinker, chiral nematic

*Corresponding authors E-mail: [svagan@kth.se](mailto:svagan@kth.se), [shanker@kth.se](mailto:shanker@kth.se)

**Table S1.** Compositions of HPC beads crosslinked with glutaraldehyde (GA).

| Entry | GA concentration  (wt%) | HPC  (mg) | GA  (mg) | 0.5 M aq. HCl  (mg) |
| --- | --- | --- | --- | --- |
| GA1 | 3 | 400 | 60 | 540 |
| GA2 | 4 | 400 | 80 | 520 |
| GA3 | 8 | 400 | 160 | 440 |

**Table S2.** Compositions of HPC beads crosslinked with borax or divinyl sulfone (DVS).

| Entry | Crosslinker concentration (wt%) | HPC  (mg) | Crosslinker | 0.1 M NaOH (mg) | Water (mg) | Remark |
| --- | --- | --- | --- | --- | --- | --- |
| B1 | 1.05 | 325 | aq. borax 175 mg | 0 | 0 |  |
| B2 | 0.6 | 325 | aq. borax 100 mg | 0 | 75 |  |
| B3 | 0.525 | 325 | aq. borax 87.5 mg | 0 | 87.5 |  |
| C1 | 0 | 325 | 0 | 55 | 120 | control 1 |
| D1 | 3 | 325 | DVS 15 mg | 55 | 105 |  |
| D2 | 4 | 325 | DVS 20 mg | 55 | 100 |  |
| D3 | 16 | 325 | DVS 80 mg | 55 | 40 |  |
| D4 | 24 | 325 | DVS 120 mg | 55 | 0 |  |
| D5 | 24 | 325 | DVS 120 mg | 0 | 180 | control 2 |

| 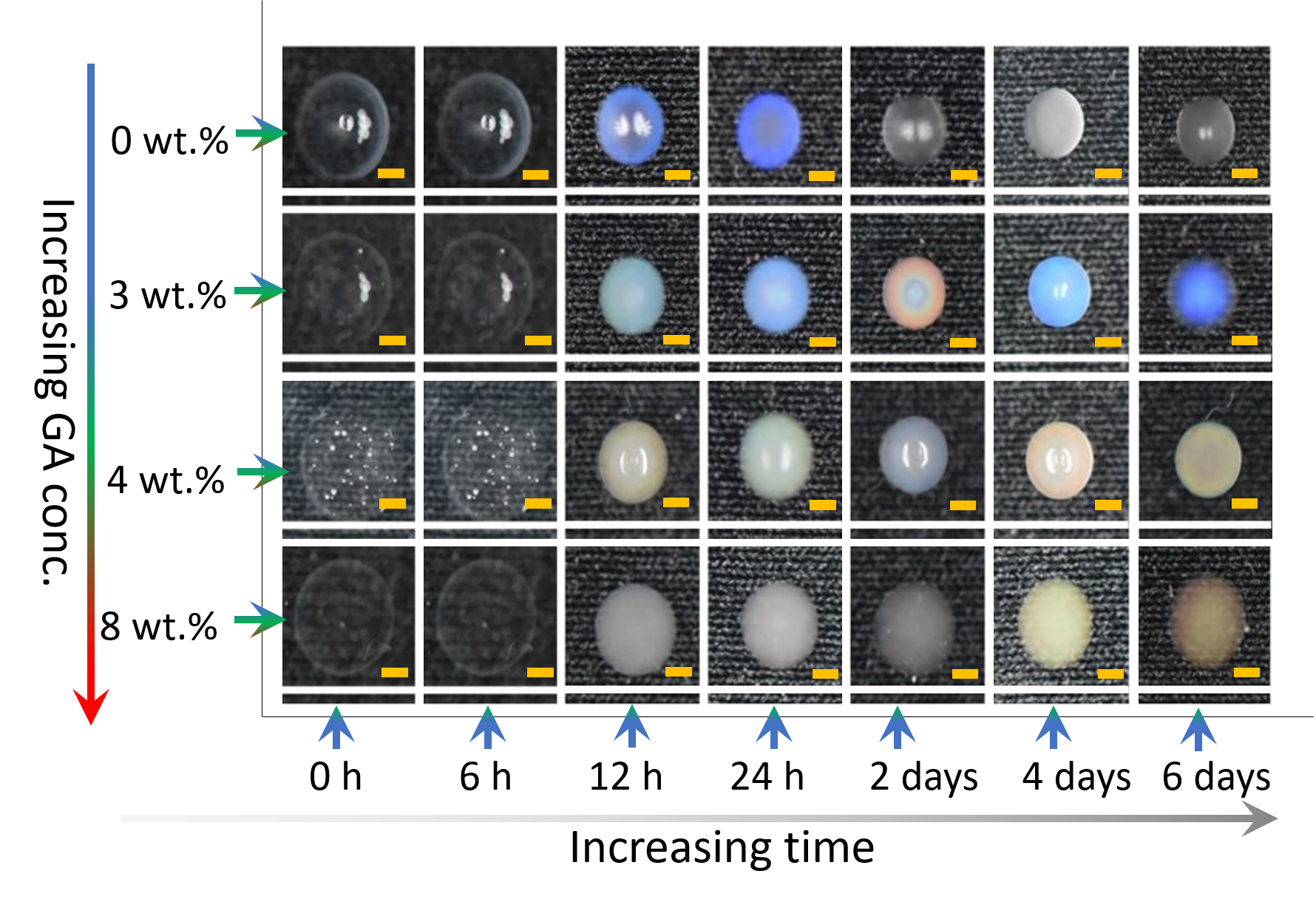 |
| --- |
| **Figure S1.** Time evolution of HCP SCBs as a function of crosslinker concentration: Photographs showing the color evolution of HPC beads on hexadecane crosslinked with glutaraldehyde. The samples were illuminated with a directional light source and imaged at angles of 0º. The scale bar is 1 mm. |

| 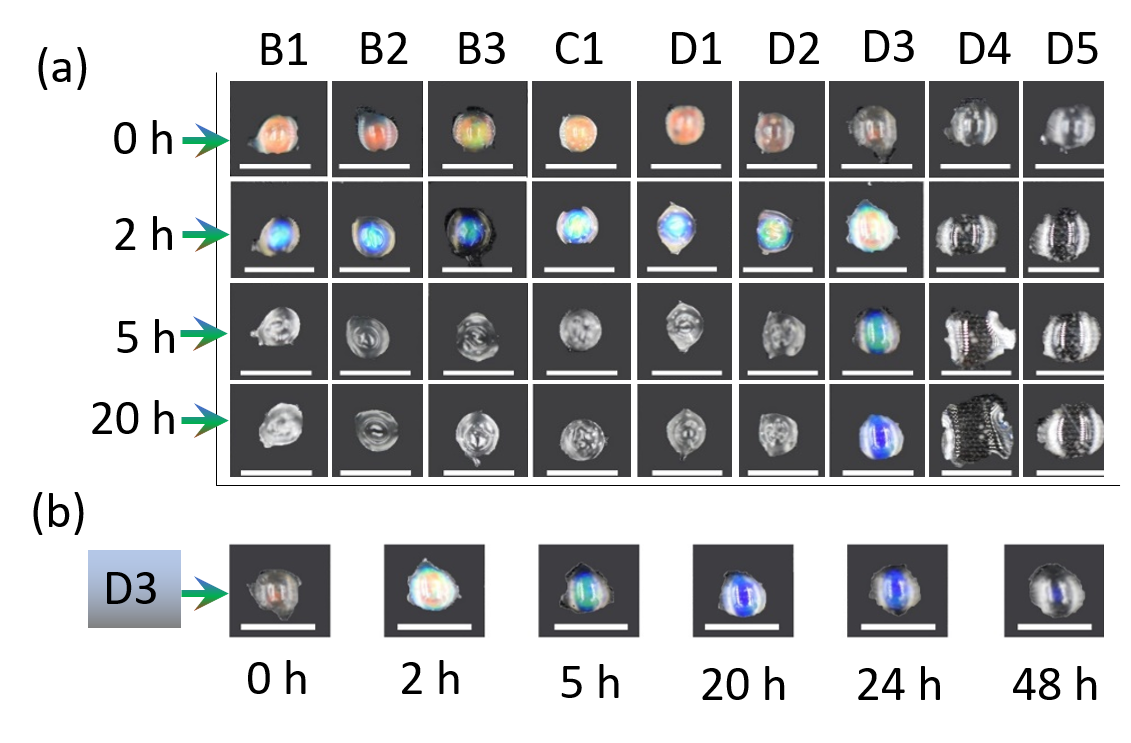 |
| --- |
| **Figure S2.** Time evolution of HCP SCBs as a function of borax or divinyl sulfone crosslinker. (a) Photographs of crosslinked HPC with borax or divinyl sulfone at different time intervals. (b) Photograph of crosslinked HPC with divinyl sulfone (sample D3) until 48 hours. The scale bar is 4 mm |

|  |
| --- |
| **Figure S3.** Reflection spectra of blue (i), green (ii), and orange (iii) SCBs, displaying multiple measurements for each sample type to demonstrate the consistency in color and spectral response across different samples of the same kind |

| 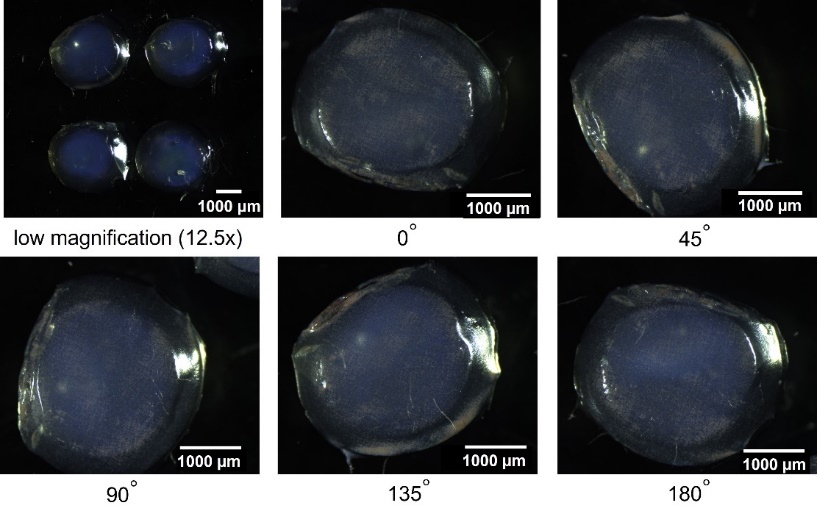 |
| --- |
| **Figure S4.** Polarized optical microscopy of blue SCBs. Optical micrograph of dried blue HPC beads recorded in transmission mode and between crossed polarizers at angles of 0°, 45°, 90°,135° and 180°. |

| 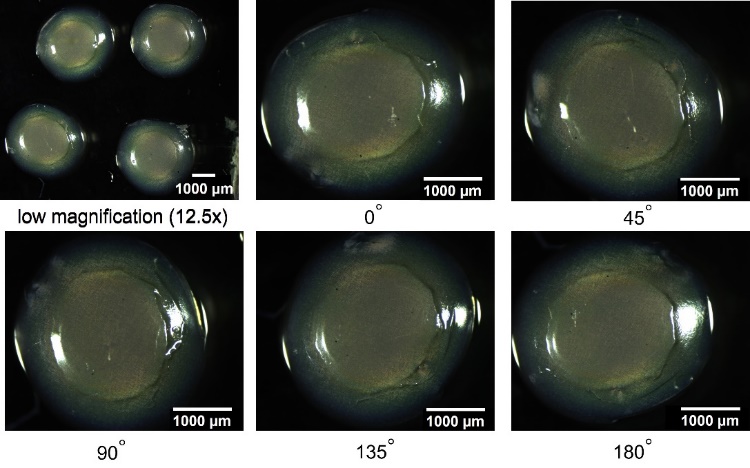 |
| --- |
| **Figure S5.** Polarized optical microscopy of green SCBs. Optical micrograph of dried green HPC beads recorded in transmission mode and between crossed polarizers at various angles. |

| 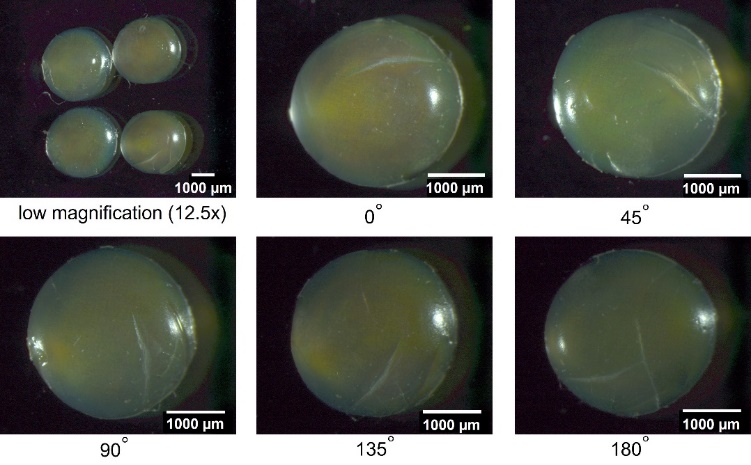 |
| --- |
| **Figure S6.** Polarized optical microscopy of orange SCBs. Optical micrograph of dried orange HPC beads recorded in transmission mode and between crossed polarizers at various angles. |
|  |
| **Figure S7.** Micro-reflection spectra of blue (i), green (ii), and red (iii) SCBs, showing measurements taken from the bulk and the edges of the beads. The spectra demonstrate a a blueshift at the edges due to different evaporation rates. (b) Corresponding microscope images of blue (i), green (ii), and red (iii) SCBs, illustrating the observed color variations between the bulk and the edges. |
| 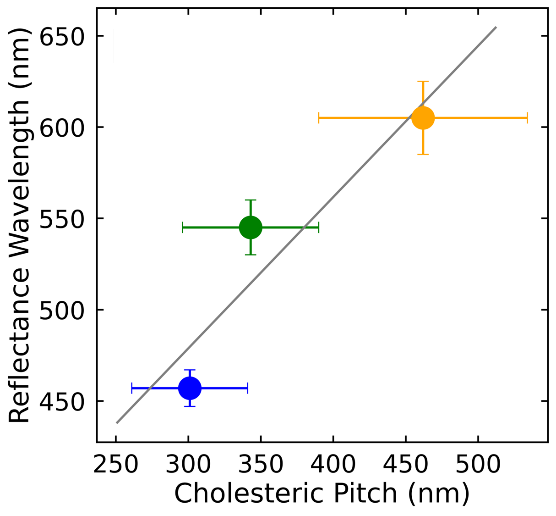 |
| **Figure S8.** Plot of cholesteric pitch (nm) against reflectance wavelength (nm), showing that the peak reflectance wavelength increases with increasing cholesteric pitch. |
| 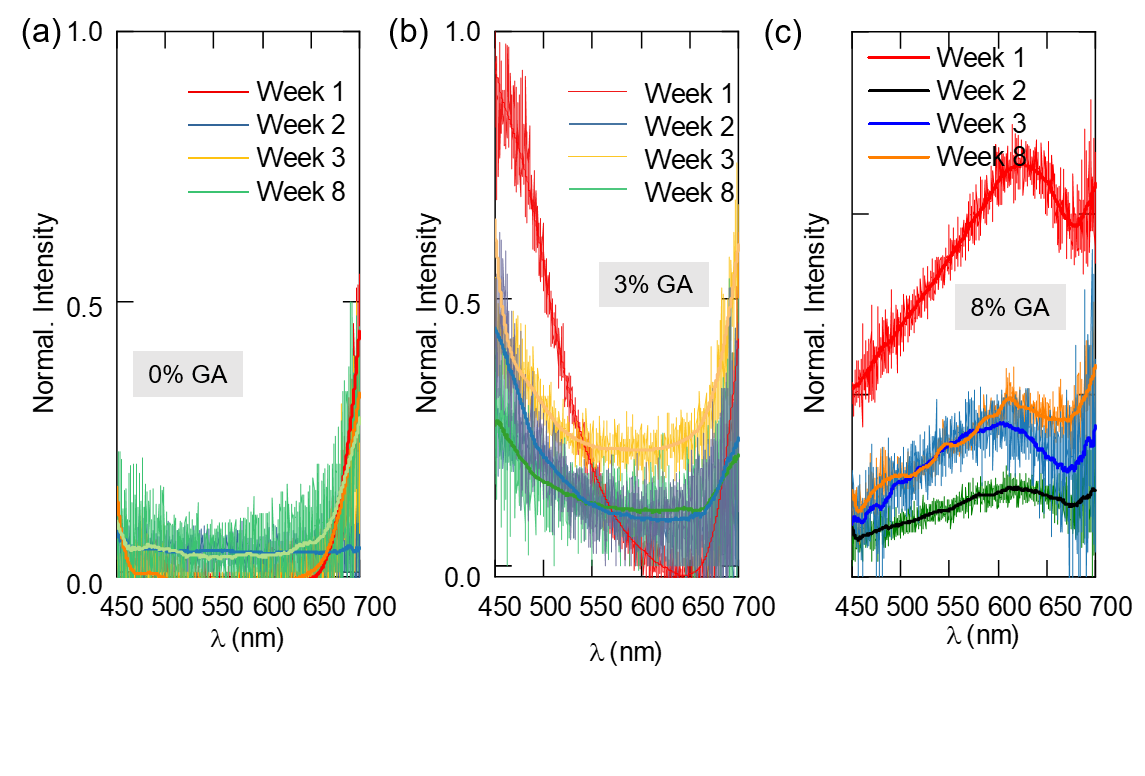 |
| **Figure S9.** Temporal stability of SCBs and nc-SCBs. Reflectance spectra of SCBs beads with crosslinker concentrations of 0%, 3%, and 8% GA over different time periods (1 week, 2, 3, and 8 weeks). Raw data (lighter, semi-transparent lines) is overlaid with smoothed lines (bold, opaque) generated using a Gaussian filter to provide clearer visualization of trends. The smoothing is applied for visual clarity and does not alter the underlying noisy nature of the raw data. The spectra show consistent reflectance peaks across all time points, demonstrating color stability. |

|  |
| --- |
| \| 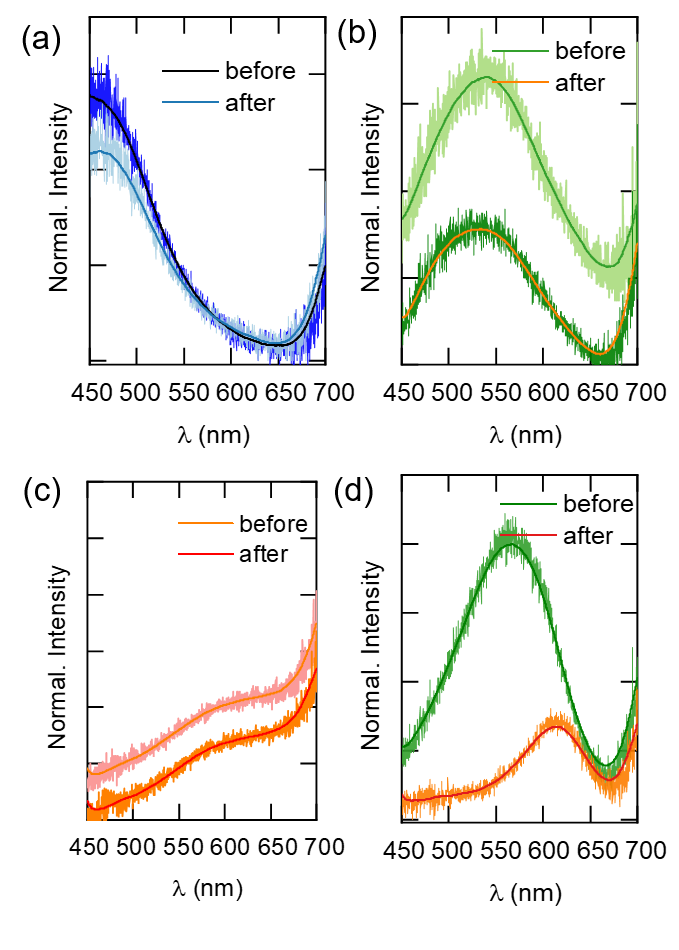 \| \| --- \| \| **Figure S10.** Effect of water immersion on optical properties of SCBs. Reflectance spectra and photographs in inset of (a) blue (b) green (c) orange crosslinked SCBs and (d) nc-SCBs before and after immersion in water. The micrographs in the inset with solid line correspond before immersion and dashed line correspond to after 5 min immersion. \| |
